# Flame-Retardance Functionalization of Jute and Jute-Cotton Fabrics

**DOI:** 10.3390/polym15112563

**Published:** 2023-06-02

**Authors:** Most Setara Begum, Abdul Kader, Rimvydas Milašius

**Affiliations:** 1 Faculty of Mechanical Engineering and Design, Kaunas University of Technology, Studentu Str. 56, LT-51424 Kaunas, Lithuania; rimvydas.milasius@ktu.lt; 2 Department of Textile Engineering, BGMEA University of Fashion and Technology, Dhaka 1230, Bangladesh; abdul_kader@buft.edu.bd

**Keywords:** jute, Jute-Cotton, flame-retardancy, TGA, free formaldehyde, Pyrovatex CP New

## Abstract

Jute is a ligno-cellulosic natural fiber that ranks second in terms of the volume of cellulosic fibers and is extensively utilized for technical textile applications. The goal of this study is to determine the flame-retardance (FR) properties of pure Jute and Jute-Cotton fabrics treated with Pyrovatex CP New at concentrations of 90% (owf), M:L: 1:7. Both fabrics exhibited a substantial improvement in flame-retardancy. After the ignition period, the recorded flame spread time in both FR treated fabrics was zero seconds; whereas for untreated Jute and Jute-Cotton fabrics, the flame spread time was measured 21 s and 28 s, respectively, to burn their entire length (15 cm). Within these flame-spread times, the length of the char was 2.1 cm and 2.57 cm in the Jute and Jute-Cotton fabrics, respectively. After FR finishing, on both fabrics in the warp and weft directions, the physico-mechanical properties significantly decreased. The deposition of flame-retardant finishes on the fabric surface was determined by Scanning Electron Microscope (SEM) images. According to Fourier Transform Infra-Red Spectroscopy (FTIR) analysis, the flame-retardant chemical had no effect on the inherent properties of the fibers. Thermogravimetric analysis (TGA) analysis revealed that FR treated fabrics had early degradation, resulting in the formation of more char than in the untreated samples. After FR treatment, both fabrics showed a significant improvement in residual mass (more than 50%). Although the formaldehyde content observed in the FR treated samples was significantly greater, it was still within the permitted limit formaldehyde content in textiles intended for outerwear and not worn next to the skin. The results of this investigation demonstrate the potential use of Pyrovatex CP New in jute-based materials.

## 1. Introduction

Jute is a ligno-cellulosic fiber that mainly consists of hemicellulose (22–24%), cellulose (58–60%) and lignin (12–14%), as well as a few other minor constituents. Due to changes in chemical composition, different components exhibit different thermal behaviors [1]. Previous studies [2,3] conducted some preliminary research on thermal behavior and fire-retardant finishes. The Jute carpet backing, decorative jute furnishing fabrics, and brattice cloth for mines have all been made fire-resistant to reduce the number of fire-related deaths and injuries. Currently, a wide range of products use FR fabrics, including floor coverings, floor mats, carpets, military uniforms, hospital furniture, hospital curtains, and industrial ventilation, among others [4]. The primary challenges associated with the fire-protective finish treatment of jute-based fabrics are higher chemical add-on, apparent tensile strength reduction, and yellowing. Furthermore, most of these FR compositions are not durable or semi-durable and require substantial dosages of pertinent chemicals [5].

The first attempts to produce fire-resistant fabric were made in England in 1735. Previous authors described the mode-of-action of FR chemicals on cellulose [6]. The FR of cotton has been extensively studied, whereas Jute has received less attention. Earlier studies using borax-boric acid with diammonium phosphate [7], potassium-sodium tartarate (Rochelle Salt) as a fire-retardant agent for Jute cited in a review study by Pal et al. [8], ammonium sulfamate (AS) with urea to improve the flame-retardancy of Cotton [9] and Jute [1]. Despite substantial drawbacks, halogenated, phosphorus-based and sulfur-based flame-retardants have been employed to improve the FR of wood [10], cotton [11], and synthetic [12] textiles.

Few investigations have been conducted on natural fibers other than cotton; however, the studies by Yusuf [13] on linen, hemp, silk & wool, while Mehta & Hoque [14] studied the FR of Jute. Dorez et al. investigated the effects of the content of cellulose, hemicellulose, and lignin on the pyrolysis and combustion of natural fibers [15]. Previous studies reported the application of different organophosphates and other chemicals in Jute [16] and in a recent study, Samanta et al. have successfully investigated the effect of nano-zinc oxide as a flame-retardant finish on Jute fabric [17] and Roy et al. reported the durable flame-retardancy in Jute [18].

The chemical composition of cellulose-based fibers differs according to their origin (that is, seed, leaf, cane, fruit, wood, bast, and grass). Most natural fibers begin to degrade at temperatures exceeding 170–200 °C. The main fiber constituents of Jute, cellulose, lignin, and hemicellulose, and each of them behave differently. Rapid pyrolysis of hemicellulose takes place between 220 and 315 °C. Lignin forms char, which protects the integrity of the textile surface by serving as a layer of fire insulation. Increased char formation during the burning process indicates flame retardancy Lignin decomposes slowly, making it harder to process, but a high cellulose content can make fibers more flammable [15]. Many scientists have examined the improvement of FR of cellulosic fibers by using different types of flame-retardant such as Ammonium Polyphosphate (APP) in different natural fibers and their biocomposites [19], Aluminium tri-hydroxide (ATH), zinc borate (ZB), melamine phosphate (MMP) and melamine borate (MMB) were applied in insulating materials made of flax short fibres [20], sodium stannate (20%) and boric acid (20%) in Jute fibers [21].

The main drawbacks of water-soluble fire retardants is the lack of durability, which led to a thorough investigation of other techniques to make fabrics robust and fire-retardant. Most of the widely used flame retardant substances today are categorized as hazardous compounds, such as halogen-based fire retardants, THPC, and APO. Numerous publications discuss the ability of cotton to retard fire; however, there are few studies that compare it to jute [5]. The potential effects of various fire retardants such as thio-urea [22], phosphorus and nitrogen based compound [3], nano zinc-oxide [17], sulfamate and urea mixture [1] on jute fabric have been previously reported. However, the majority of these treatments require high temperature curing. Due to the lack of adequate equipment in most of jute mills for high-temperature curing operations, protective finishing procedures without curing are needed for industrial applications. However, it frequently affects the mechanical [23] and comfort [24] properties of the textile product and can even be allergic. Therefore, it is essential to select the most appropriate treatment and chemical concentration of flame retardant.

Due to their strong flame resistance, halogenated flame retardants (such as polychlorinated biphenyls and decabromodiphenyl ethers) are often used in cotton fabrics [25]. However, halogen-based flame retardants contaminate the environment and pose health risks when they are burnt [26], producing toxic and corrosive chemicals [27]. As a result, halogenated flame retardants were gradually banned by the European Union [28]. The most prominent phosphate-based flame retardants on the market today are Proban and Pyrovatex CP [29]. Recent studies have shown that several environmentally friendly techniques, including the use of nanoscale metal oxides and biomolecules derived from plants and animals, are effective substitutes for traditional fire-retardant formulations [30].

Pyrovatex, unfortunately, has the problem of releasing formaldehyde and additionally requires formaldehyde-based cross-linkers to enhance the fire retardancy, durability, and easy-care performance. As a result, the treated cotton fabric contains substantially more harmful formaldehyde. Formaldehyde is a well-known inexpensive and effective cellulose cross-linker. Sadly, formaldehyde is associated with major health risks, including eye and skin irritation, headaches, breathing difficulties, and most importantly, it is a known carcinogen to humans [31,32].

This study intends to apply a flame-retardant finish to 100% Jute and Jute-Cotton fabrics treated with Pyrovatex CP New, a commercially available phosphorus-based flame-retardant (FR), and a formaldehyde-free cross-linking agent KNITTEX FFRC. Thereby we analyzed the thermal degradation properties through Thermogravimetric Analysis (TGA) and burning studies. This FR chemical is mainly commercialized for cotton. In this study, the same FR chemical has been applied to pure Jute and Jute-Cotton mixed fabrics, as there is no prior published work stating the use of this flame-retardant on these materials.

## 2. Materials and Methods

### 2.1. Materials

All experiments were carried out using two different plain-woven fabrics consisting of 100% Jute (290 GSM, EPI: 22, PPI: 16) and Jute-Cotton (Warp: Cotton, and Weft: Jute; 340 GSM, EPI: 52, PPI: 26), purchased from Mony Jute Company, Dhaka, Bangladesh.

A phosphorous-based commercial flame-retardant chemical, PYROVATEX CP New (N-methylol dimethylphosphonpropionamide) and the cross-linking agent KNITTEX FFRC (a modified dihydroxyethylene urea) were purchased from the local agent of Huntsman (SwissColours Bangladesh Limited, Dhaka, Bangladesh). The chemical formula of both chemicals is presented in Table 1. All chemicals were used as received.

### 2.2. Fabric Pretreatment

Before flame-retardant finish treatment, to remove impurities, both fabrics were scoured and bleached using Caustic soda (100 g/kg), hydrogen peroxide (80 g/kg), at a liquor ratio of 1:10. The pretreatment process was run for 30 min at 50 °C. Finally, the treated fabrics were neutralized using acetic acid for 10 min.

### 2.3. Flame-Retardant Finishing Treatment

100% Jute and Jute-Cotton fabrics were treated with PYROVATEX CP NEW 90% (owf), KNITTEX FFRC crosslinking agent was used 30 g/L at material-to-liquor ratio 1:7. In an open bath at room temperature, the fabric samples were immersed in the solution for 1 h. Phosphoric acid (25%) was used to control pH (3.5–6). Finally, the treated samples were dried in the air.

### 2.4. Flammability Test

The untreated and FR treated fabrics were tested using a vertical flammability test. The specimen size was kept 15 cm in length and 5 cm in width and exposed to a standard flame at 90° vertically using a Bunsen burner for 10 s of ignition time; the flame source is then removed and left for further burning. Afterwards, the flame spreading time, after glow time and char length was recorded. All tests were repeated three (03) times.

### 2.5. Physico-Mechanical Properties Analysis

The effect of the flame-retardant finish on the physico-mechanical properties of the treated fabrics was determined by the tensile strength test, the tearing strength test, and the elongation at break. For the tensile strength test and the elongation at the break, the maximum force was determined using the grab method (EN ISO 13934-2). The tear force was determined using the single tear method (EN ISO 13937-2(auto-stop)). All tests were repeated three (03) times.

The *add*-*on*% of treated samples were measured by using Equation (1) and expressed as a percentage.

(1) $$Add-on\;\left(\%\right)=\frac{W_{2}-W_{1}}{W_{1}}\times 100$$

where, $W_{1}$ and $W_{2}$ indicate the oven-dry weight of the fabric samples before and after FR treatments, respectively. 

### 2.6. Surface Characterization

The surface topography characterization of the fabric samples was examined using a scanning electron microscope (SEM, SU 1510, Hitachi, Japan) using the Carbo filament technique without coating. An Attenuated Total Reflectance Fourier Transform Infra-Red (ATR-FTIR) mode (Diamond ATR, resolution 4 cm^−1^, scan number 16, range of analysis 4000–500, JASCO 4700, Japan) was used to study changes in chemical composition by FR treatment on both fabrics.

### 2.7. Thermal Properties Analysis

Thermogravimetric Analysis (TGA) was performed under a nitrogen atmosphere with a NETZSCH (STA 449 F3 Jupiter, Erich NETZSCH GmbH & Co. Holding KG, Selb, Bayern, Germany) Thermogravimetric Analyzer Instrument. A 10 mg sample was heated from room temperature to 600 °C at 10 °C/min.

### 2.8. Formaldehyde Test

The free formaldehyde content on the Jute and Jute-Cotton fabric samples was determined by using ISO 14184-1, analyzed using the UV-VIS Spectrophotometer (Lambda 25, Perkin Elmer, UK). Formula to calculate the amount of formaldehyde extracted for each sample (*wF*), to the nearest mg/kg, using Equation (2):

(2) $$wF=\frac{\rho \times 00}{m}$$

where, *ρ* is the concentration of HCHO in solution, in mg/L, as read in the calibration graph, *m* is the mass of the specimen, in grams.

## 3. Results and Discussion

### 3.1. Determination of Flammability

According to the findings of our previous investigation [33], the treatment condition was optimized at a 1:7 material-to-liquor ratio for a 90% (owf) concentration of Pyrovatex CP New in Jute fabrics. Apparently, the Jute-Cotton fabric was treated with the same parameters, and the performance was measured.

**Figure 1:**
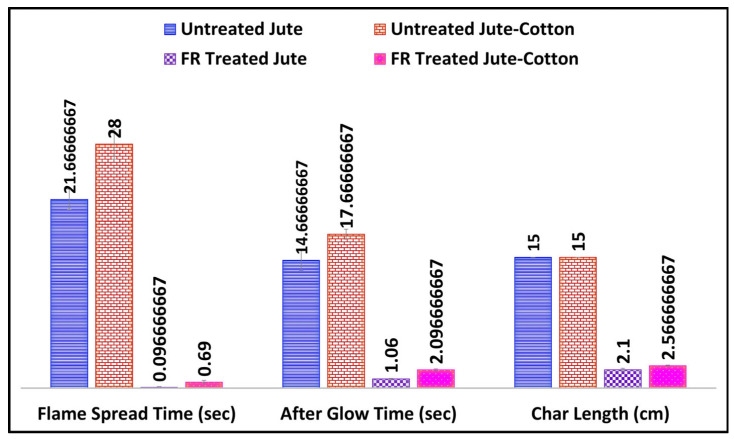
Flammability Properties of Jute and Jute-Cotton Fabrics (M:L: 1:7, Pyrovatex CP New: 90% owf).

**Figure 2:**
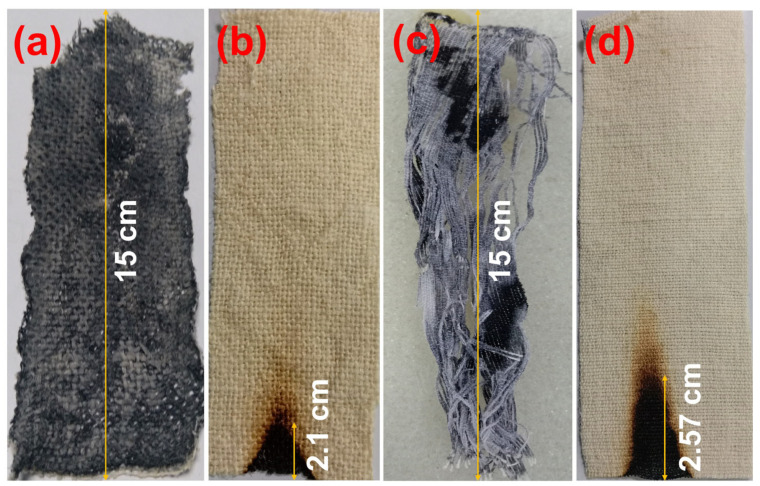
Digital images of Char formation after vertical flammability test of (**a**) Untreated Jute Fabric, (**b**) FR Treated Jute Fabric, (**c**) Untreated Jute-Cotton Fabric and (**d**) FR Treated Jute-Cotton Fabrics.

Figure 1 and Figure 2 represent the vertical flammability properties and digital images of 90% Pyrovatex CP New (1:7) treated Jute and Jute-Cotton fabrics, respectively. Both treated fabrics exhibited good FR ability in terms of flame-spread time and char length. It was observed that after the flame source was removed, the flame spread time was less than one second on the FR treated Jute-Cotton fabric, whereas it was zero on the FR treated Jute fabric. The afterglow time on 100% Jute fabric remained zero, while it was two seconds for the Jute-Cotton fabric. Similarly, a slightly higher char length (2.57 cm) was determined on the Jute-Cotton fabric than on 100% Jute (2.1 cm). On the contrary, both untreated fabrics were entirely (15.0 cm) burned spreading the flame for 21.6 s and after glowing for 14.6 s on pure jute and 28 s and 17.6 s, respectively, on the Jute-Cotton fabrics (Figure 1). These findings show a significant improvement in FR in the Jute-based materials used in this study and therefore meet the requirements for the ability to FR of decorative fabrics (B1 rating, ≤5 s) in support of the previous findings [34,35].

### 3.2. Reaction Mechanism of the FR Chemical, Knittex FFRC and Cellulose

**Figure 3:**
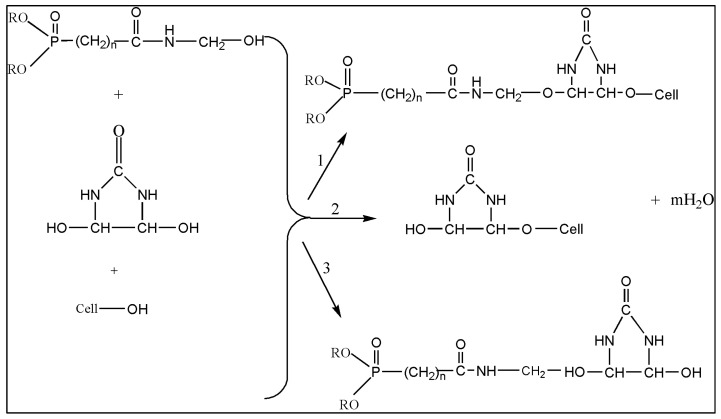
Crosslinking mechanism of Knittex FFRC with Pyrovatex CP New and cellulose [36,37].

There are several scenarios that can occur. A chemical mode-of-action is provided in reaction 1 of Figure 3 to show the effective crosslinking by Knittex FFRC between Pyrovatex CP New and cellulose. Additionally, Knittex FFRC may only react with cellulose (reaction 2) or with Pyrovatex CP New (reaction 3). Based on the flammability results presented above, it is possible to anticipate that any of these reactions may occur or at least occur to some extent; nevertheless, it is difficult to claim which reaction actually took place throughout this treatment process. This statement agrees with the findings of earlier observations [36,37].

### 3.3. Physico-Mechanical Properties Analysis

**Figure 4:**
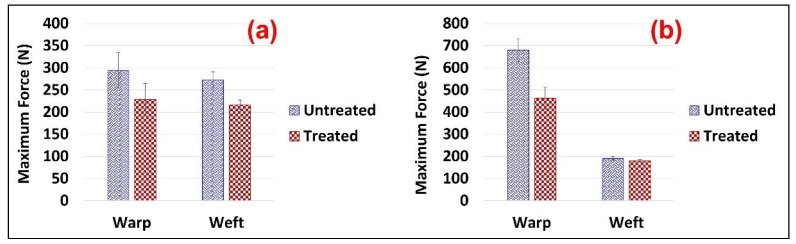
Tensile Strength of (**a**) Jute and (**b**) Jute-Cotton Fabrics.

The chemicals add-on% on the FR treated samples was 21.6% and 28.8% for the Jute and Jute-Cotton fabrics, respectively. This indicates the excellent bonding between the Pyrovatex CP New and the cellulose structure in the presence of Knittex FFRC cross-linking. This phenomenon might occur because Knittex FFRC has two-OH groups that can create links with cellulose. The extent of physico-mechanical damage was determined through the loss of tensile and tear strength and elongation at break measurements. The results of the tensile strength tests for the Jute and Jute-Cotton fabrics in the warp and weft directions are shown in Figure 4. The figure shows that the tensile strength of the FR-treated fabrics drastically decreased in both directions compared to the untreated fabrics. About 23% strength loss was observed in the warp direction of the Jute fabrics, while about 21% loss was observed in the weft direction of the same fabrics. On the contrary, the maximum tensile strength loss of about 32% was found in the warp direction on Jute-Cotton fabrics, while a comparatively small loss (7%) was observed in the weft direction.

These results correspond to a greater strength loss in Jute than in cotton by flame-retardant treatment, since a little strength loss was found in the weft direction of the Jute-Cotton fabric. A similar finding was reported earlier [38]. This reduction in tensile strength can be attributed to the use of a cross-linking agent in the flame-retardant finish bath.

**Figure 5:**
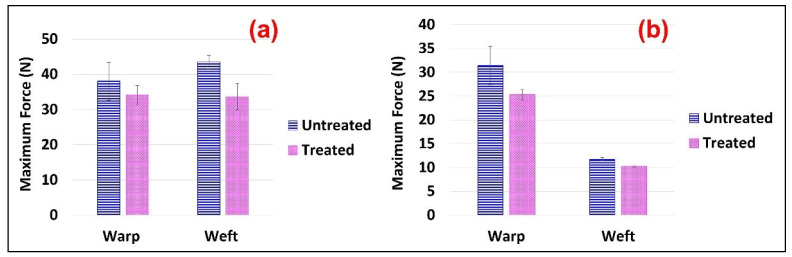
Tear Strength of (**a**) Jute and (**b**) Jute-Cotton Fabrics.

Similarly, the tear strength loss was determined in the FR-treated Jute fabrics in both the warp and weft directions. Although the trend of strength loss remains almost similar (Figure 5a) to the tensile; however, it was more than twice in the weft direction (23%) than in the warp (10%). On the other hand, a significant loss in tear strength was observed in the warp direction (19%) of the Jute-Cotton fabric after FR treatment, which is nearly double that of Jute fabrics in the same direction. A 13% strength loss was observed in the weft direction of the same fabric (Figure 5b). Consequently, a similar tear strength loss trend was seen in the warp direction of Jute and the weft direction of Jute-cotton fabrics and vice versa. These findings are consistent with previous studies [24,38]. This happens as the yarns adhere to each other due to the cross-linking agent in the flame-retardant finish, which reduces the mobility of the yarn and tearing strength of the yarn [39].

**Figure 6:**
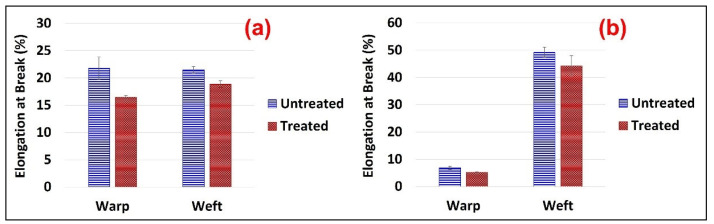
Elongation at Maximum Force of (**a**) Jute and (**b**) Jute-Cotton Fabrics.

Figure 6 illustrates the elongation at maximum force applied to the untreated and FR treated fabrics. A significant loss of elongation was observed on the FR treated fabrics in both the warp and weft directions. The maximum loss of elongation (24%) was observed in the warp directions of both fabrics, whereas almost 12% and 10% were seen in the weft directions of the Jute and Jute-Cotton fabrics, respectively. Variations in the yarn used in both directions may be the cause of differences in the initial and final elongation loss in the warp and weft directions of Jute-Cotton fabrics. The technical cause of this elongation loss in the FR treated fabric may be related to variations of the twist set in the yarns and the subsequent crimp on each set of yarns generated from the compactness of the weave structures used in both fabrics. Furthermore, FR-treated fabrics may exhibit less elongation due to enhanced cohesiveness between fibers and between yarns, which is consistent with the previous study [24].

### 3.4. Surface Chemical Composition and Surface Morphology Analysis

**Figure 7:**
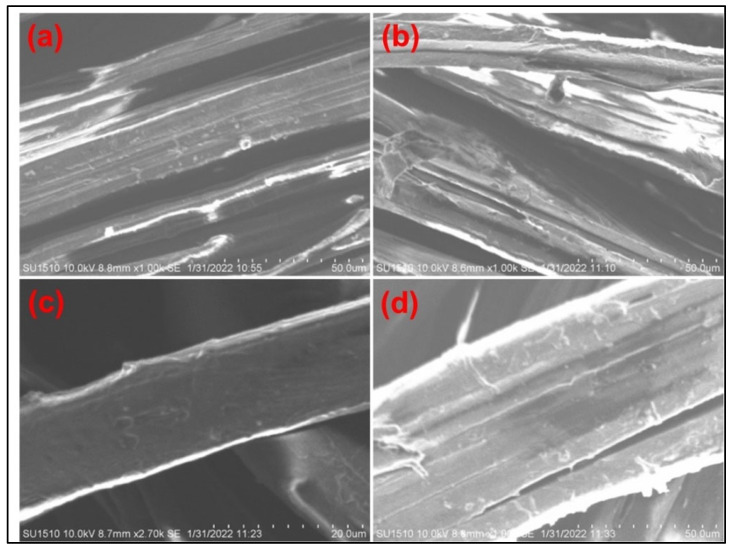
SEM Images of (**a**) Untreated Jute, (**b**) FR Treated Jute; (**c**) Untreated Jute-Cotton and (**d**) FR Treated Jute-Cotton Fabrics.

The surface morphology and homogeneous distribution of flame-retardant coatings on the Jute and Jute-Cotton fabrics were inferred from the Scanning Electron Microscopy (SEM) images in Figure 7. The FR performance of the treated fabrics is influenced by the homogeneity of the FR chemical deposition on the fabric surface. Greater flame-retardance is frequently indicated by the fact that the chemicals on the surface are more homogeneous. As seen in the figure, the untreated samples had a smooth surface, whereas the images of both FR treated samples exhibit some irregular and rough surfaces. This indicates that during the treatments, FR chemicals were deposited on the surface of the fiber.

**Figure 8:**
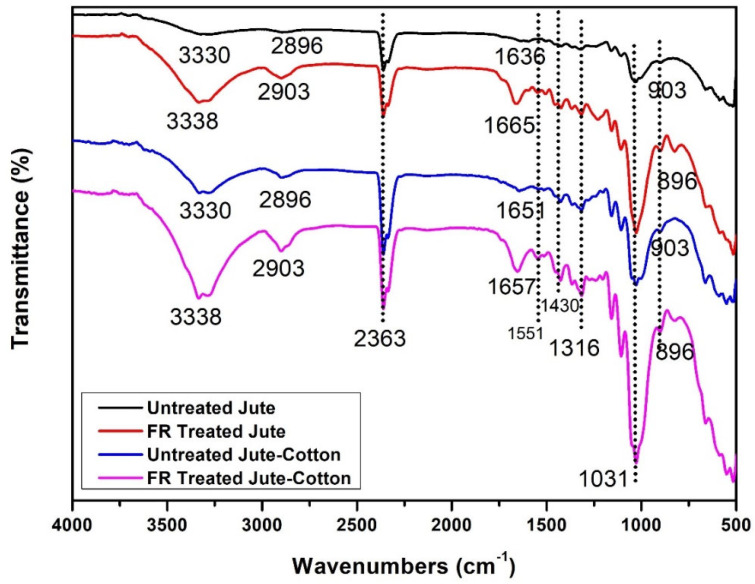
FTIR results of Untreated and FR Treated Jute and Jute-Cotton Fabrics.

The chemical composition changes of untreated and FR treated pure Jute and Jute-Cotton fabrics are illustrated in Figure 8. ATR-FTIR analysis was used to determine the surface chemical composition of the substrate. These spectra are concentrated in the 4000 to 500 cm^−1^ spectral range. The peaks at 3330, 3338, 3330, and 3338 cm^−1^ in the case of untreated Jute, FR treated Jute, untreated Jute-Cotton, and FR treated Jute-Cotton, respectively, represent hydrogen-bonded (OH) stretching; this is one of the distinctive bands of the spectrum associated with the α-cellulose in fiber [40]. Additionally, the enlarged peak areas at 3330–3338 cm^−1^ for the vibration of the CH-OH stretching, the stretching vibration of KNITTEX FFRC and Pyrovatex CP New, as well as the holding H_2_O molecules in FR treated fabrics, are responsible for the increased hydrophilicity of FR treated fabrics. along with. Like this, the peaks for C-H stretching vibrations of aliphatic methylene groups were found at 2896 and 2903 cm^−1^ for both untreated and FR-treated Jute and Jute-Cotton fabrics, respectively. These peaks represent the presence of the aldehyde group; the existence of CH and CH_2_ in cellulose and hemicellulose was shown by stretching and bending C-H [41]. At 2896–2903 cm^−1^ and for increased C-H and CH_2_ stretch vibrations for KNITTEX FFRC along with Pyrovatex CP New also merged with C-H and CH_2_ stretch vibrations of cellulose in Jute and Jute-Cotton in the same wave numbers at 3330–3338 cm^−1^. The peak was apparent at 2363 cm^−1^ and was associated with the stretching of C-H in polysaccharide chains [1]. The gradual increase in the height of the peak at 2363 cm^−1^ is due to the additional vibration of the amide group (-CO-NH) present in Pyrovatex CP New as obtained after FR treatment and also due to -C-N- stretching for amino group of cross-linking agent molecules (KNITTEX FFRC) added in FR treatment. The visible peaks of both the FR treated substrates at 1665 and 1657 cm^−1^ are shifted from their untreated substrates at 1636 and 1651 cm^−1^, corresponding to the water molecules (H-O-H group) of natural fibers [42], as well as the carbonyl groups (C=O) present in lignin and hemicellulose [43]. The visible peaks in this region found on FR treated fabrics are due to the presence of Dimethyl Ester moieties of Pyrovatex CP New. The carbonyl stretching modes of the carboxylate anion cause a new peak at 1551 cm^−1^, which is attributed to the carbonyl bond (C=O) [44]. These spectra demonstrate that a flame-retardant chemical was present in the samples. The inherent symmetric bending of CH_2_ in lignin is related to absorbance at 1430 cm^−1^ [45] and centered on wagging CH at 1316 cm^−1^ [24]. The sharp peak centered at 1031 cm^−1^ was associated with the C-O group of the hydroxyl and ether groups present in the cellulose [24,41]. Furthermore, the increase in peak height at 1030–1031 cm^−1^ can be viewed as the increase in -OH and CH-OH wagging cellulose intensifies with the stretching vibration of H_2_O (water) molecules held by amide-ester of phosphate compounds (Pyrovatex CP New) as FR chemical applied to Jute and Jute-Cotton fabrics. Peaks at 896 and 903 cm^−1^ of untreated and FR treated specimens, respectively, indicated β-glucosidic linkage and were attributed to O-C-O stretching during the C-H deformation of cellulose [46].

### 3.5. Thermal Properties Analysis

**Figure 9:**
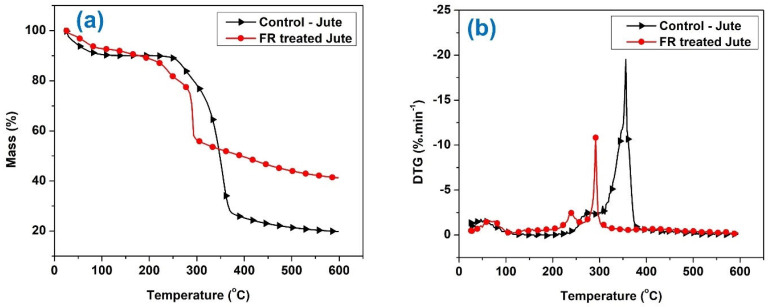
TGA curves (**a**) and DTG (**b**) curves of Jute textiles under nitrogen.

**Figure 10:**
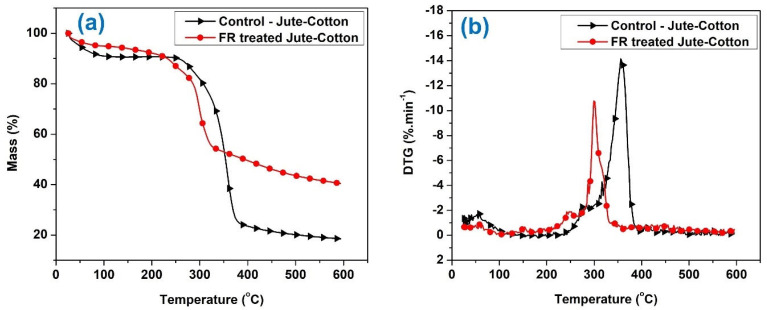
TGA curves (**a**) and DTG (**b**) curves of Jute-Cotton textiles under nitrogen.

Figure 9a,b and Figure 10a,b show the TGA and DTG curves for untreated and flame-retardant treated jute and jute-cotton fabrics, respectively. For untreated fabrics, the main pyrolysis and rapid weight loss stage is observed at temperatures around 360 °C. This stage is associated with the dehydration and decarboxylation reactions that produce more combustible gases; and the temperature region around 400 °C corresponds to the decomposition of char formed during the pyrolysis stage [47]. A small weight loss was observed in the low temperature region, which may have been caused by the moisture present in the samples. According to earlier studies, the amorphous region of the polymer where cellulose is mainly degraded during the primary stage of pyrolysis [48]. In the second stage, cellulose undergoes pyrolysis in the crystalline region of the polymers, resulting in a significant weight loss from the sample. Glucose and various types of combustible gases are the main pyrolysis products formed in this stage [48]. Studies reported that thermal decomposition of cotton produces combustible and noncombustible volatiles [49]. At high temperatures, dehydration and decarboxylation, along with the discharge of water, carbon dioxide, and carbonyl, led to a continuous reduction in mass loss [50].

In untreated Jute, hemicellulose degrades at around 250–290 °C and cellulose degrades around 360–365 °C and lignin degrades at 425 °C. For the untreated jute and Jute-Cotton fabrics in Figure 9 and Figure 10, it has a rate of change of weight loss at around 290–300 °C, which after 350 °C abruptly drops to a higher weight loss at around 350–370 °C indicating cellulose degradation, and thereafter it continues weight loss at a different rate and continues up to 450 °C before stopping for no further weight loss. This minute weight loss in between 425 to 450 or 500 °C before no further weight loss point is reached at a slower rate is degradation of the lignin part. For FR treated Jute and Jute-Cotton fabrics, there is early degradation of all three major constituents of Jute. It shows that hemicelluloses of FR treated Jute begin and end their thermal degradation at 200 to 250 °C and continue to start the thermal degradation of the cellulose part at 250 to 300 °C; however, at a different abrupt rate that results in a faster weight loss but quickly but larger residue. According to weight loss data, lignin of FR treated Jute degrades at temperatures 350–400 °C and at above, but at a significantly slower rate of thermal degradation that continues up to 450 °C by producing more char and leaving behind more residual weight.

According to the DTG curves, the main decomposition peak for untreated Jute occurs at 356 °C. Figure 9 and Figure 10 show that FR treated Jute went through a similar pyrolysis stage, but the decomposition peak shifted to lower temperature at 291 °C. The early thermal degradation can be due to phosphoric acid that is generated during the pyrolysis of phosphorous-containing compounds present in the flame-retardant chemical. This acts as a dehydrating agent leading to a lower decomposition temperature and a higher residual char yield. Similarly, for untreated Jute-Cotton fabrics, the main pyrolysis and decomposition stage is observed at 356 °C; whereas, the decomposition temperature is observed at 299 °C for FR-treated Jute-Cotton fabrics. However, the initial decomposition temperature for FR treated fabrics was also reduced from 257 °C to 215 °C and from 266 °C to 217 °C for Jute and Jute-Cotton fabrics, respectively.

**Figure 11:**
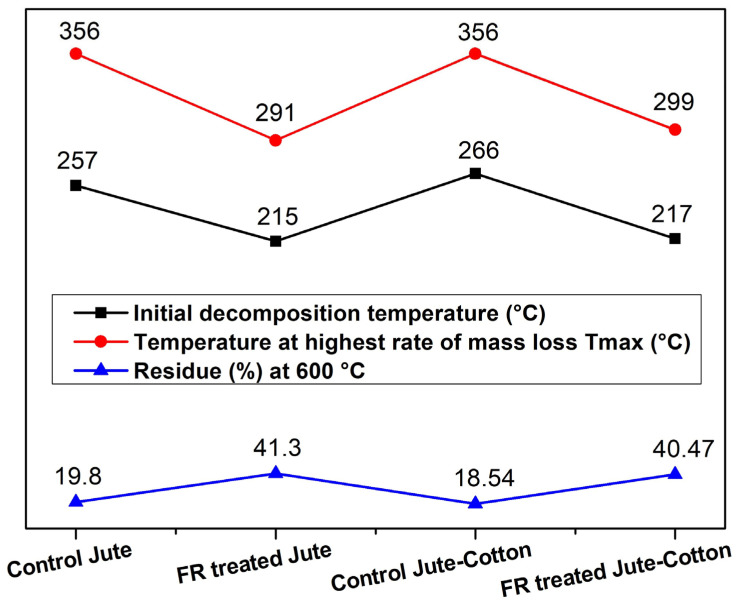
Thermogravimetric Analysis Data of Jute and Jute-Cotton Fabric Samples.

The initial degradation temperatures, temperature at highest rate of mass loss and final char yields at 600 °C are presented in Figure 11. The figure shows that the FR treated Jute fabric produced a higher amount of char residue (41.30%) at 600 °C than the untreated Jute (19.80%). A higher char residue at 600 °C is also observed for FR treated Jute-Cotton fabrics (40.47%) than that of untreated Jute-Cotton fabrics (18.54%). The residuary weights of these samples are almost constant above 378 °C and 306 °C for untreated Jute and FR treated Jute fabrics, whereas they were 391 °C and 340 °C for untreated and FR treated Jute-Cotton fabrics, respectively. These results indicate a significantly higher char formation in the FR treated fabrics than in their untreated pairs. This occurs because of FR treatment, which altered the pyrolytic behavior of cellulosic fabrics and resulted in the formation of char of cellulosic fibers prior to pyrolysis [51]. According to earlier research, greater FR performance is correlated with an increase in char formation in these temperature regions [52]. These results of thermal analysis clearly express the significant improvement in flame-retardancy in FR treated fabrics [23].

### 3.6. Formaldehyde Content Analysis

**Figure 12:**
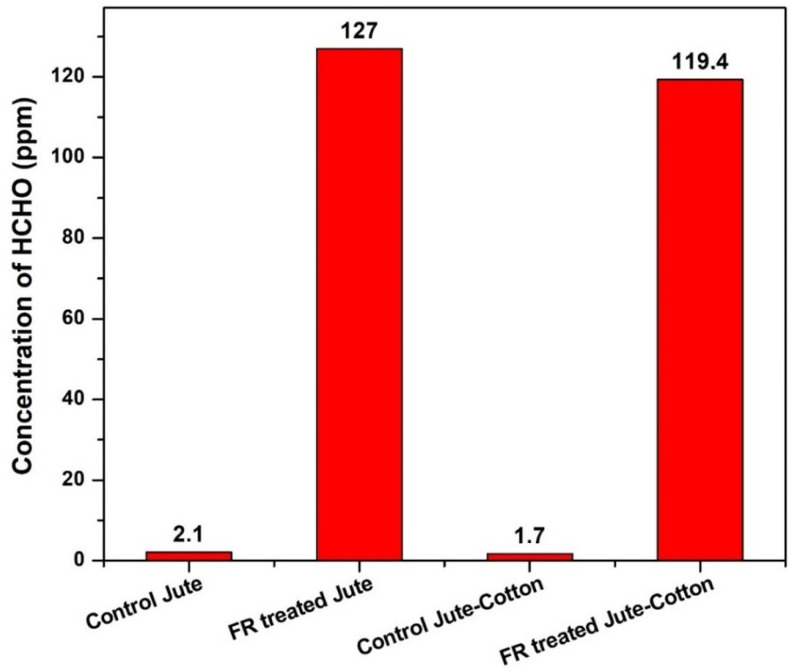
Formaldehyde content measured in the samples.

Formaldehyde was designated as Group 1 carcinogen for humans by the International Agency for Research on Cancer (IARC) in 2004 based on toxicological data and epidemiological evidence obtained in workplaces [53,54]. One of the most debilitating dermatological conditions is clothing-related allergic contact dermatitis. Formaldehyde-containing resins have been employed in the clothing industry to produce wrinkle-resistant fabrics since 1926 [55]. According to previous reports, formaldehyde release levels in industrial practise have dropped from 2000 ppm to 100–200 ppm in the US textiles [56,57]. However, it was not clearly mentioned which specific product was chosen for this observation. Nevertheless, it can be presumed that the textiles in that study could fall into the category of outerwear textiles. In this study, after FR treatment, the fabric samples were kept at room temperature for a week. Subsequently, the free formaldehyde measured in the samples in this study was 127 ppm and 119.4 ppm for the FR-treated Jute and Jute-Cotton fabrics, respectively (Figure 12) and could correspond to the FR chemical used in this work. However, a negligible content of formaldehyde is also found in the untreated samples, which may be due to external contamination. Pyrovatex CP New was the primary source of free formaldehyde, supporting Drago Katovic’s prior findings that cotton fabrics treated solely with Pyrovatex CP New without crosslinking may possibly contain free formaldehyde at levels greater than 300 ppm [31,58]. The findings of this study meet the requirements of textiles that are not in direct contact (e.g., outerwear). The European Ecolabel (Commission Decision 2002/371/EC) and the private Oeko-Tex Standard 100 are two voluntary labelling schemes that consider ecological and consumer protection criteria. In both schemes, the limit for textiles not in direct contact with the skin (e.g., outerwear) and decorative materials is 300 mg/kg. Ecolabel established a limit of 30 mg/kg for textiles in direct contact with the skin, whereas Oeko-Tex Standard 100 established a limit of 75 mg/kg. Furthermore, Oeko-Tex Standard 100 established a limit for baby textiles that should emit less than 20 mg/kg of formaldehyde [59,60]. Recent research work emphasizes more on developing the ecofriendly FR chemicals and the formaldehyde free crosslinking and binding agents that are required for the successful FR functions. Some studies suggested the use of a non-methylol substitute for the organophosphorus agent, plant-based FR chemicals such as banana pseudostem sap [61], ammonium polyphosphate (APP) and branched polyethyleneimine (BPEI) [62], phytic acid [63] silicon–phosphorus–nitrogen synergistic flame retardant [26].

## 4. Conclusions

The current study explores the potential to use a readily accessible flame-retardant chemical to enhance the FR properties of Jute and Jute-Cotton fabrics. The thermogravimetric findings indicate early decomposition in both the FR treated Jute and Jute-Cotton fabrics. The significant amount of residual mass as reported more than 50% in FR treated fabrics indicates thermal stability; however, the treatment also caused a notable loss of physical strength. The FR treated fabrics showed better char-forming ability during the thermal degradation and combustion stage. The tensile strength and tear of the FR-treated fabrics decreased significantly in both the warp and weft directions. SEM images showed some rough and irregular surfaces in the morphological stage of the fabric, indicating that the surface deposition of flame-retardant chemicals on the surface of the fiber occurred during the treatments. Despite the detected formaldehyde content on FR treated fabrics, it still meets the permitted formaldehyde content present in textiles for external use and is not worn directly next to the skin.

Firstly, the Pyrovatex CP New was chosen for this study because of the commercial availability developed for cotton materials. Since Jute fiber constitutes around 60% cellulose in its chemical composition along with other constituents. To assess the potential for FR performance, Pyrovatex CP New was applied to Jute and Jute-Cotton fabrics. These FR treated materials can be used for manufacturing products such as curtains, upholstery, bio-degradable welding gloves, etc. when frequent washing is not necessary. Despite the slight differences in the char length, higher tensile strength loss was observed in the warp direction of the FR treated Jute-Cotton fabrics than in the pure Jute; nevertheless, the strength loss appears to be opposite in the weft direction. The least loss of strength and elongation was determined in the weft direction of Jute-Cotton fabrics compared to pure Jute. This finding gives the indication of higher strength of Jute than Cotton, as the Jute was used in the weft direction of Jute-Cotton fabrics.

Lignin in Jute tends to create more char during burning, and char acts as an insulation and inhibits the propagation of the fire. On the contrary, it also exhibits the harsh and brittle nature of Jute fiber. The presence of lignin on both fabrics was indicated by FTIR and the degradation nature of lignin was explained by TGA analysis in this study. To overcome the limitation, and therefore to improve the handle along with the flame-retardant property of fabric, a Jute-Cotton combination fabric can act as an influential alternative. In addition, Jute fiber is cheaper, and the cultivation process is also cleaner than that of cotton. The consumption of chemicals, pesticides, and water in cotton production is higher, whereas Jute production is associated with minimal uses of chemicals and pesticides. Furthermore, Jute grows during the monsoon season; therefore, no external water is needed during the cultivation. Nevertheless, Jute is still facing challenges to achieve commercial success for potential applications. Therefore, with respect to the environmental aspect of these cellulosic fibers, the jute fiber is more sustainable and promising to meet the requirements for technical applications.

## Figures and Tables

**Table 1:** Chemical name and formula.

Chemical Name	Chemical Formula
Pyrovatex CP New	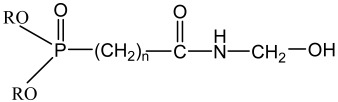
Knittex FFRC (dihydroxyethylene urea)	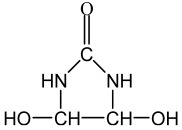

## Data Availability

Not applicable.
